# Treatment of *Clostridium difficile* infection in community teaching hospital: a retrospective study

**DOI:** 10.1186/s40545-020-00289-1

**Published:** 2021-02-11

**Authors:** Ali Elbeddini, Rachel Gerochi

**Affiliations:** 1 Winchester District Memorial Hospital, 566 Louise Street, Winchester, ON KK0C2K0 Canada; 2 Leslie Dan Faculty of Pharmacy, University of Toronto, 144 College St, Toronto, M5S 3M2 Canada

**Keywords:** *Clostridium difficile* infection, Metronidazole, Vancomycin, Algorithm, Adherence, Infectious Diseases Society of America 2010 *C. difficile* guideline

## Abstract

**Objectives:**

*Clostridium difficile* infection (CDI) is responsible for 15–25% cases of health-care-associated diarrhea. The CDI treatment algorithm used at our hospital is adapted from the Infectious Diseases Society of America 2010 *C. difficile* guideline. The primary objective of this study was to assess the treatment adherence to our algorithm; this was defined as therapy consisting of the appropriate antibiotic, dose, route, interval, and duration indicated based on the disease severity and episode within 24 h of diagnosis. Furthermore, our study also described the population and their risk factors for CDI at our hospital.

**Methods:**

This was a single-centre, retrospective cohort chart review of CDI cases that were diagnosed at admission or during hospitalization from June 1st, 2017 to June 30th, 2018. Cases were identified by a positive stool test along with watery diarrhea or by colonoscopy.

**Results:**

Sixty cases were included, of which adherence to our algorithm was 50%. Overall, severe CDI had the highest treatment non-adherence (83%), and the biggest contributing factor was prescribing the wrong antibiotic (72%). In severe CDI, which warrants vancomycin monotherapy, wrong antibiotic consisted of metronidazole monotherapy (55%) or dual therapy with metronidazole and vancomycin (45%). Patients were mostly older, females being treated for an initial episode of mild-to-moderate CDI. Common risk factors identified were age over 65 years (80%), use of antibiotics (83%) and proton pump inhibitors (PPI) (68%) within the previous 3 months. The use of a PPI in this study, a modifiable risk factor without a clear indication, was 35%.

**Conclusion:**

An area for antimicrobial stewardship intervention in CDI treatment at our hospital is prescribing the right antibiotic based on the CDI indication. In severe CDI, an emphasis should be on prescribing vancomycin monotherapy as the drug of choice. PPI use should be reassessed for tapering when appropriate.

## Introduction

### Background

*Clostridium difficile* is an anaerobic, spore producing, gram-positive bacterium that is transmitted fecal-orally [1]. Hospitals are associated with a higher risk of transmission due to environmental contamination, the frequent use of antibiotics, and poor hand hygiene practices. Antibiotics disrupt the microflora of the colon allowing *C. difficile* to grow in high concentrations [2]. As a result, CDI rates tend to be higher between the months of November to March due to increased respiratory infections requiring use of antibiotics [3]. The rate of recurrence after an initial episode of CDI is 6–25% and increases with recurrent episodes of CDI [1, 4]. It can be manifested either by another infection caused by the original strain of *C. difficile* or a new infection caused by a new strain of *C. difficile*, while the microflora in the colon is returning to normal levels [1]. Since September 2008, *C. difficile* infections (CDI) are one of several monthly patient safety indicators reportable to the Ontario Ministry of Health and Long-Term Care (MOHLTC) under the Public Hospitals Act [3].

Although all antibiotics can contribute to CDI, clindamycin, third-generation cephalosporin and fluoroquinolones are associated with the highest rates [2]. Other medications that also carry a high CDI risk include gastric acid suppressing agents such as proton pump inhibitors (PPIs) and histamine-2 receptor antagonists (H2RAs) as they promote an environment suitable for *C. difficile* bacteria to survive [2]. Immunosuppressing medications such as antineoplastics or steroids decrease the immune system’s ability to produce antibodies and are associated with CDI as well [3]. Further risk factors associated with an increased exposure to *C. difficile* and antibiotic use include gastrointestinal surgery, irritable bowel disease, diabetes, cardiovascular, respiratory or kidney disease, patients over 65 years, use of nasogastric tubes, prolonged hospitalization or exposure to long term care facilities [5, 6].

Common symptoms of CDI include watery diarrhea, nausea, abdominal pain and fever [1]. If left untreated, CDI can, in rare instances, result in pseudomembranous colitis, toxic megacolon and death [3]. Therefore, minimizing prolonged durations of antibiotic therapy and antibiotic use without appropriate indications, and switching from intravenous to oral therapy when possible to shorten hospital stay will help decrease the risk of developing CDI [2]. Similarly, appropriate treatment of CDI based on the severity of the infection will help decrease the risk of developing a recurrent CDI [2].

At Winchester District Memorial Hospital (WDMH), CDI is diagnosed by a positive stool detection of both *C. difficile* antigen and *C. difficile* toxin A and B. If only one of the two is positive, a positive polymerase chain reaction (PCR) test for *C. difficile* toxin B is required to confirm diagnosis [7]. If the detection is negative for both antigen and toxins, or if the PCR test is negative, no CDI diagnosis is made [7]. In addition, the passage of three or more unformed stools defined by Bristol stool type 6–7 (Table 1) in 24 h must also be present to confirm the diagnosis of CDI, except patients diagnosed with an ileus or toxic megacolon [5]. Prior to initiating treatment in symptomatic patients with positive laboratory findings, the classification of the severity of CDI must be established (Table 2). To distinguish between severities, other additional signs and symptoms must be considered. Mild and moderate CDI is defined as having signs and symptoms including fever, increased abdominal pain, signs of dehydration such as decreased urine output, and leukocytosis with a WBC < 15 000 cells/ μL [8]. Severe CDI is defined as having signs and symptoms including fever, severe abdominal pain, signs of sepsis such as confusion, tachycardia and decreased urine output, acute renal dysfunction (defined as an elevated serum creatinine greater than 1.5 times the premorbid level) and leukocytosis with WBC ≥ 20 000 cells/ μL [8, 9]. Similarly, severe and complicated CDI is defined as having symptoms of severe CDI in addition to either having an ileus, toxic megacolon, shock such as a drop in systolic blood pressure [8].

**Table 1:** Bristol stool chart [14]

Type 1		Separate hard lumps, like nuts (hard to pass)
Type 2		Sausage-shaped but lumpy
Type 3		Like a sausage but with cracks on its surface
Type 4		Like a sausage or snake, smooth and soft
Type 5		Soft blobs with clear-cuts edges (passed easily)
Type 6		Fluffy pieces with ragged edges, a mushy stool
Type 7		Watery, no solid pieces, entirely liquid

Classification of stools from types 1 to 7 based on appearance and characteristics

**Table 2:** Classification of disease severity [1, 12]

Severity	Signs and symptoms
Mild and moderate	WBC < 20 000 cells/μL SCr < 1.5 times the premorbid level
Severe	WBC ≥ 20 000 cells/ μL SCr ≥ 1.5 times the premorbid level Hypotension
Severe and complicated	Ileus Toxic megacolon

Classification of CDI severity according to the signs and symptoms

Despite guidelines outlining approaches to treat CDI, many institutions are still reporting that inappropriate treatment regimens are still being given patients, ultimately leading to poorer health outcomes. This has been shown in multiple published studies outlining that guideline compliance is poor, especially in more severe CDI cases. The aim of this study is to establish the current practices and approaches in the treatment of CDI at WDMH and to identify areas, where treatment, particularly treatment adherence to guidelines, may be improved.

### Guidelines recommendations

There are three guidelines available for the treatment of *C. difficile*: The Infectious Diseases Society of America (IDSA) published in 2010, the American College of Gastroenterology (ACG) published in 2013, and the European Society of Clinical Microbiology and Infectious Diseases (ESCMID) published in 2014. All three guidelines recommend similar therapies for different treatments of CDI with minor differences such as prolonging duration of therapy and adding alternative therapies to their recommendations (Table 3) [1, 5, 10].

**Table 3:** Current antibiotic treatment recommendations for CDI [1, 5, 10, 12]

	IDSA (2010)	ACG (2013)	ESCMID (2014)	WDMH (2017)
*Initial episode of CDI*				
Mild/moderate	Metronidazole 500 mg po q8h × 10–14 days	Metronidazole 500 mg po q8h × 10 days (If no improvement in 5–7 days, consider change to severe CDI treatment)	Metronidazole 500 mg po q8h × 10 days	Metronidazole 500 mg po q8h × 10–14 days (If no improvement in 5 days or clinical worsening, change to CDI severe treatment)
Severe	Vancomycin 125 mg po q6h × 10–14 days	Vancomycin 125 mg po q6h × 10 days	Vancomycin 125 mg po q6h × 10 days	Vancomycin 125 mg po q6h × 10–14 days (If no response or symptoms worsening, consult Internal Medicine)
Severe and complicated	Vancomycin 500 mg po q6h and metronidazole 500 mg iv q8h and (If ileus present: add vancomycin 500 mg in 100 mL NS pr q6h)	Vancomycin 125 mg po q6h, 500 mg in 500 mL saline as enema pr q6h and metronidazole 500 mg iv q8h		Vancomycin 125–500 mg po q6h and metronidazole 500 mg iv q8h (Consider vancomycin pr or immunoglobulin iv)
1st recurrent episode of CDI	Same treatment as initial episode of CDI	Repeat metronidazole or vancomycin pulse regimen	Vancomycin 125 mg po q6h × 10 days	Same treatment as initial episode of CDI
2nd recurrent episode of CDI	Vancomycin in a tapered and/or		Fecal transplant combined with oral antibiotic	Vancomycin 125 mg po q6h × 10–14 days
	pulsed regimen		treatment	(Consider consulting Internal Medicine and a vancomycin tapering regimen or pulsed regimen i.e. vancomycin 125 mg po qid × 7 days, then 125 mg po bid × 7 days, then 125 mg po daily × 7 days, then 125 mg po q2d × 7 days then 125 mg po q3d × 14 days then discontinue)
Pregnant or patients intolerant to metronidazole	No recommendations	Vancomycin 125 mg po q6h × 10 days	No recommendations	Same treatment as severe CDI

First-line antibiotic regimens recommended by current international guidelines in comparison to WMDH guidelines

For treatment of initial episodes of mild-to-moderate CDI, the general consensus from all three guidelines is metronidazole 500 mg PO q8h for 10–14 days [1, 5, 10]. The ESCMID guidelines also recommends vancomycin 125 mg PO q6h for 10 days as an alternative to metronidazole [5]. The ACG recommends switching therapy to treatment for severe CDI if there is no improvement after 5–7 days with the current therapy [10].

For treatment of initial episodes of severe CDI, the general consensus from all three guidelines is vancomycin 125 mg PO q6h for 10–14 days [1, 5, 10]. Another alternative the ESCMID recommends is to consider increasing the vancomycin dose to 500 mg q6h for 10 days [5]. For treatment of initial episodes of severe, complicated CDI, the general consensus from all three guidelines is vancomycin 125–500 mg PO q6h ± metronidazole 500 mg IV q8h [1, 5, 10]. In the presence of an ileus, the IDSA recommends adding vancomycin 500 mg in approximately 100 mL normal saline PR q6h as a retention enema [1]. In an ileus, toxic megacolon or abdominal distension, the ACG recommends triple therapy consisting of vancomycin 500 mg in a volume of 500 mL PR q6h, vancomycin 500 mg PO q6h and standard iv metronidazole therapy [10].

For the first recurrent episode of CDI, all the guidelines recommend following the same treatment as for an initial episode of CDI [1, 5, 10]. In the second or multiple recurrent episodes of CDI, the general consensus to avoid peripheral neuropathy with metronidazole, is vancomycin 125 mg PO q6h for 10–14 days followed by either a pulse or taper regimen [1, 5, 10]. The intermittent dosing that follows the scheduled vancomycin dosing continues to suppress levels of *C difficile* while allowing the microflora of the colon to return to normal [10].

In addition to the pharmacological treatment for CDI, non-pharmacological options such as the use of fecal transplant or surgery may be indicated in specific cases of recurrent CDI [11]. Incorporation of infection control and prevention techniques such as proper hand hygiene, environmental disinfection and single room isolation will also help minimize the spread of *C. difficile* [1]. The role of probiotics is still uncertain in the prevention or treatment of CDI and is currently not part of the treatment algorithm [1]. Finally, other pharmacological treatment approaches to increase the efficacy of treatment involves discontinuing current laxatives and acid suppressing medications, if possible, since they worsen symptoms of CDI and increase the risk of recurrent CDI, respectively [12]. The IDSA and ACG guidelines also recommend avoiding antidiarrheal medications, since they increase the retention of *C. difficile* toxins and the risk for toxic megacolon.

### Current situation

At WDMH, the *C. difficile* treatment algorithm is adapted from the The Ottawa Hospital (TOH) *C. difficile* treatment algorithm (Table 3). Both hospital algorithms are based on the IDSA 2010 guidelines. For this research project, the two main antibiotics included will be metronidazole and vancomycin, since the new antibiotic, fidaxomicin and fecal transplant are not included in the WDMH Treatment algorithm.

For treatment of initial episodes of mild-to-moderate CDI, the WDMH algorithm recommends metronidazole 500 mg PO q8h for 10–14 days [12]. If there is no improvement or if there is significant clinical deterioration at day 5, therapy should be escalated for treatment of severe CDI, which is vancomycin 125 mg PO q6h for 10–14 days [12]. In the presence of ileus or toxic megacolon, dual therapy of vancomycin 125–500 mg PO q6h and standard iv metronidazole therapy with the consideration of administering vancomycin 500 mg in the form of an enema in 100 mL normal saline (NS) for 60 min q4–12 h or intravenous immunoglobulin is recommended [12]. Treatment for first recurrent episode of CDI remains the same as initial episode of CDI. Treatment for second or multiple recurrent episodes of CDI is vancomycin 125 mg PO q6h for 10–14 days followed by either a pulse or taper regimen [5, 12]. In patients who are pregnant or who are intolerant to metronidazole, the WDMH algorithm recommends vancomycin 125 mg PO q6h for 10–14 days [12].

### Extending duration of CDI treatment with concomitant antibiotics

Patients who receive antibiotics for other infections during treatment of CDI or when the colon microflora has not returned to normal are at an increased risk of recurrent CDI. Since the disruption of the colon microflora can lasts for days and up to weeks after completion of therapy, some clinicians continue treatment of CDI until the antibiotic therapy is completed [1]. However, it is unknown whether this practice reduces the risk of a recurrent CDI [1]. Contrary, the ACG guidelines state that there is no evidence to support the continuation of CDI treatment in patients who are also on non-CDI antibiotics [10]. Therefore, the evidence and consensus regarding the duration of *C. difficile* therapy is lacking. There is, however, expert opinion-based recommendations that in patients receiving concurrent antibiotics for other infections, treatment for *C. difficile* should be continued for at least 7 days after the completion of non-CDI antibiotics [13]. Depending on the number of episodes, the duration of extended CDI therapy may be adjusted to correlate with the associated risk of recurrence. As a result, at WDMH, although it is not part of the treatment algorithm, it is recommended to continue *C. difficile* treatment for a minimum of 7 days in patients who have completed non-CDI antibiotics [12].

### Primary research objectives

The first primary objective of this study is to describe the CDI treatment adherence at WDMH to the WDMH *C. difficile* treatment algorithm, which is based on the IDSA 2010 *C. difficile* guidelines. Adherence to treatment algorithm is defined as the appropriate antibiotic, dose, route, interval, duration, time to start and stop dates of antibiotics indicated based on the classification of CDI severity. The second primary objective is to describe the population and their risk factors for CDI at WDMH.

### Secondary research objectives

1. Describe the current practice at WDMH regarding continuation of CDI treatment after completion of non-CDI antibiotics used to treat other infections.
2. Describe the rate of recurrent CDI associated with the duration of CDI therapy after the completion of non-CDI antibiotics used to treat other infections.

## Methodology

### Study type

This research project was a single-centre, retrospective cohort study.

### Study sample and inclusion/exclusion criteria

**Fig. 1:**
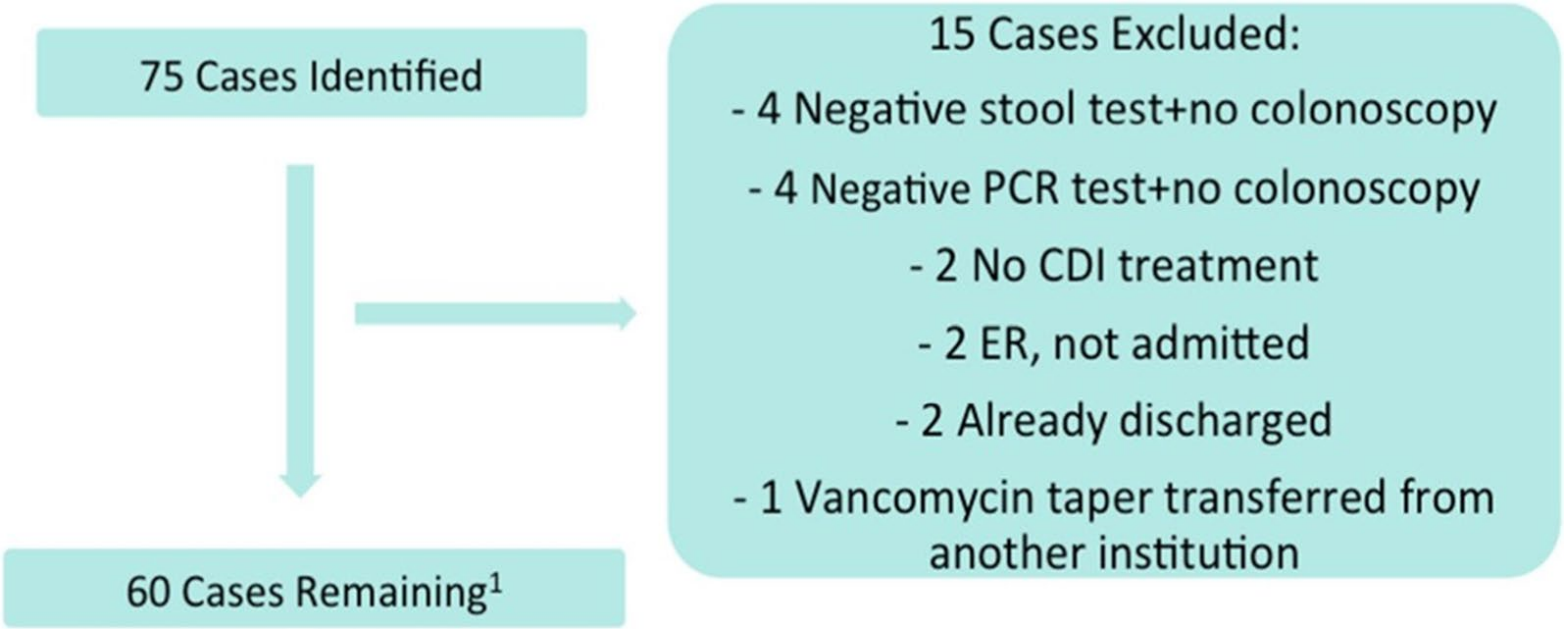
Flow diagram of case selection. Of a total of 75 possible cases identified, 15 cases were excluded for the above reasons. Following their exclusion, 60 cases were examined for the study

The primary inclusion criterion for the study is either an initial or recurrent CDI episode diagnosed at admission or during hospitalization at WDMH from June 1st, 2017 to June 30th, 2018. Recurrent CDI episodes are defined as episodes following a previous occurrence in the last 8 weeks, where the initial episode was resolved with appropriate treatment. CDI diagnosis was determined by a positive stool test for C. *difficile* antigen, toxin A and B (or positive PCR test for toxin B if necessary when stool toxin is not positive) along with the passage of three or more unformed stools, defined as Bristol Scale Type 6–7 (Table 1) in 24 h. Alternatively, a colonoscopy detecting pseudomembranous colitis was also used to determine diagnosis. Patients with negative results or those who did not undergo confirmatory testing were, therefore, excluded from the study. Other reasons for exclusion include cases not admitted and those in which a full course of CDI treatment was not initiated and completed during the patient’s hospitalization at WDMH. Including patient cases in which the entirety of CDI treatment can not be fully observed and assessed can negatively affect the accuracy of the study’s outcomes. 75 cases were identified during the observation period that were documented as potential CDI cases, 60 of which satisfied the inclusion and exclusion criteria of the study. Figure 1 outlines the number of patient cases that were excluded and their specified reasoning for exclusion. These 60 cases were based on 56 patients in total as two patients had two episodes of CDI each and were therefore accounted for twice.

Sample size refers to the number of CDI cases rather than the number of CDI patients. A sample size range of 50 to 100 was chosen based on data availability from June 1, 2017 to June 30, 2018 and time limitations to complete this project within a year. This range is reasonable as the main goal of this study was largely descriptive and data was exclusively gathered from a single site. WDMH has averaged about 0.28 CDI cases per 1000 patient days since 2017, according to Health Quality Ontario, further supporting the relatively smaller sample size compared to larger institutions that may see more cases. Therefore, a sample size range between 50 and 100 cases was enough to provide reliable descriptive statistics that can also be used for areas of improvement.

### Data collection

Two different sources were used to collect data including QuadraMed and patient records. QuadraMed is an enterprise master patient index (EMPI) software used at WDMH that compiles patient data together from various sources to provide consistent patient information. Quality control for 10% of the CDI cases were reviewed by Primary investigator. From these sources, data was collected on Bristol stool classification 48 h prior to discharge to determine CDI resolution. If a patient showed no loose stools or no Bristol Types 6 or 7, 48 h prior to their discharge, their CDI episode was deemed as resolved. Secondary outcome data such as 30-day all-cause mortality, 30-day readmission to WDMH and recurrence of CDI within 8 weeks of a previous episode was also collected.

Treatment adherence was measured by comparing each patient’s medication regimen recorded in their patient profile to the WDMH CDI treatment algorithm adapted from the IDSA guidelines. Discrepancies in the form of wrong drug, dose, route, and duration were deemed as treatment that was not adherent.

### Data analysis

All data collected were entered and analysed with Microsoft Excel. The primary objective pertaining to treatment adherence to our algorithm, based on the IDSA 2010 *C. difficile* guidelines was expressed as nominal data as a percentage of yes or no. The analyses of breakdown of different components were based on the disease severity. If the wrong drug were prescribed, that would automatically be counted, as non-adherent and no further analyses would be done. If a regimen contained the right drug, further investigation regarding appropriate dose, route, interval and duration were analyzed. Description of population and their risk factors was expressed using descriptive statistics including the calculation of the mean and median, as applicable. The secondary objective, continuation of CDI treatment after completion of non-CDI antibiotics, was expressed as continuous data based on the number of days therapy was continued. The association between the rates of recurrent CDI and duration of CDI therapy after the completion of non-CDI antibiotics was expressed as nominal data as a percentage of yes or no categorized by the days of CDI therapy was continued into either less than 7 days or 7 days or more.

Overall outcomes including CDI resolution defined as no loose stool (Bristol Type 6–7) within 48 h prior to discharge, 30-day all-cause mortality, 30-day readmission and 8-week CDI recurrence were analyzed based on receiving adherent and non-adherent treatment and were reported using the odds ratio test. Statistical significance was defined as having a p-value < 0.05.

Treatment adherence was based on the CDI treatment initiated and disease severity listed in the algorithm including hypotension defined as SBP < 90 mmHg and measured white blood cell (WBC) count within the 24 h of diagnosis.

## Results

Of 75 potential CDI cases identified between June 1st, 2017 and June 30th, 2018, 60 cases had a confirmed diagnosis of CDI and a subsequent full course of antibiotic treatment observed at WDMH. The characteristics of each patient were collected and summarized in Table 4 to obtain an average representation of the population. The most prevalent risk factors for CDI seen in the population was age > 65 years and at least one prescribed antibiotic prior to the CDI episode (Table 5). These findings are consistent with the risk factors identified by IDSA guidelines.

**Table 4:** Baseline characteristics

	Average ± SD
Age (years)	75 ± 15
Body mass index (kg/m^2^)	21 ± 14
Length of stay [3] (days)	12 ± 24
	*# Out of 60 (%)*
Female	38 (63)
Episode:	
Initial	55 (92)
1st Recurrence	4 (7)
2nd Recurrence	1 (2)
Severity:	
Mild to moderate	34 (57)
Severe	24 (40)
Severe and complicated	2 (3)

Average characteristics of the study population

**Table 5:** Risk factors

	# Out of 60 (%)
Age ≥ 65 years	48 (80)
Length of stay ≥ 14 days	11 (18)
Clindamycin [1]	2 (3)
Fluoroquinolone [1]	30 (50)
3rd Generation Cephalosporin [1]	20 (33)
At least one antibiotic [1]	50 (83)
Proton pump inhibitor [1]	41 (68)
Histamine-2 receptor antagonist [1]	5 (8)
Hospitalization [3]	23 (38)
Recurrent CDI [5]	5 (8)

Risk factors for CDI identified in the study population

**Fig. 2:**
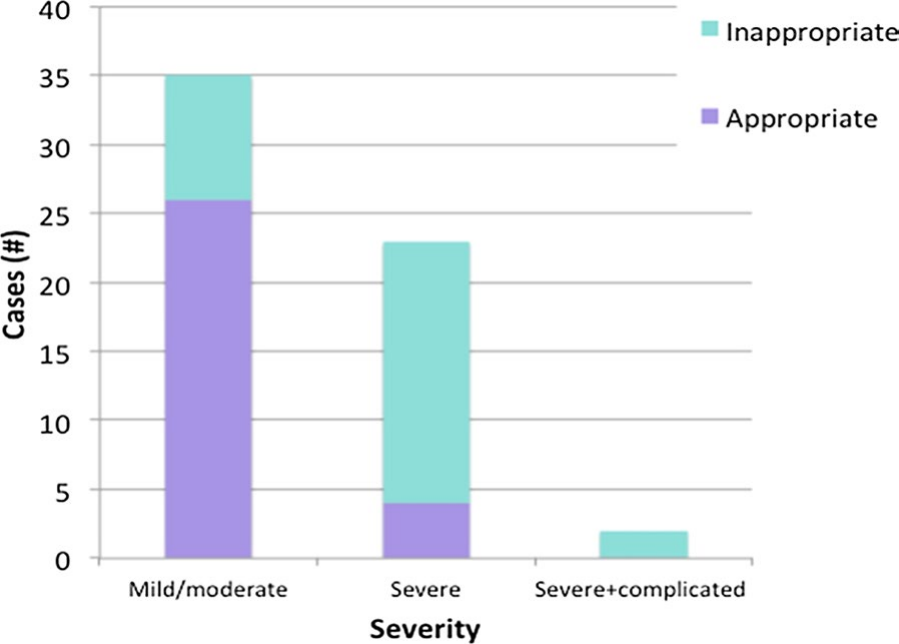
Treatment appropriateness by severity. As the severity of the CDI cases increased, the proportion of cases that received inappropriate treatment also increased. Of those classified as severe and complicated, none were treated appropriately

Adherence based on disease severity is shown in Fig. 2 and suggest that CDI treatment adherence to our algorithm has areas for improvement. The most common severity was mild-to-moderate a treatment adherence of 74%. Treatment for this severity with metronidazole 500 mg PO q8h every 10–14 days is consistent across guidelines and the WDMH algorithm. This is not the case for severe or severe and complicated CDI cases, where the classification is not as straight forward, and treatment depends on clinician expertise and experience. The treatment regimens recommended by IDSA for severe and severe and complicated cases are consistent with our algorithm—vancomycin 125 mg PO q6h and metronidazole 500 mg IV q8h in combination with vancomycin 125–500 mg PO q6h, respectively. Severe cases had the highest rate of non-adherence at 83%. As the classification of the signs and symptoms in this category can differ, treatment prescribed for patients may vary depending on whether physicians use the IDSA guideline or WDMH algorithm classification. This may explain the low adherence to treatment. The severe and complicated disease severity is rare at WDMH with only two cases, both of which were non-adherent to treatment.

Patients that received the appropriate treatment had higher rates of CDI resolution compared to those than received inappropriate treatment (p = 0.001) (Table 6). Only 58% of cases were deemed resolved. This can be due to death from other reasons or early discharge, classifying the case as unresolved. Patients that received inappropriate treatment also experienced a higher rate of 30-day all-cause mortality (p = 0.007). Non-adherent therapies in certain situations can resolve CDI similar to adherent therapies. For example, non-adherent treatments for mild-to-moderate CDI such as vancomycin or dual combination therapy are still also effective at resolving CDI. When interpreting 30-day all-cause mortality, it is important to keep in mind that many patients had concomitant infections along with CDI, and whether the cause of death due to a specific infection or in combination with CDI, or for a complete other reason is unknown. More patients who received appropriate treatment had higher rates of 30-day readmissions and 8-week recurrence. It is difficult to draw conclusions from these outcomes as there are other factors that can affect recurrence such as proper cleaning of patient rooms. Whether they were re-infected or developed a new CDI during hospital or after discharge is unknown and would both count towards recurrence.

**Table 6:** Adherent treatment and overall outcomes

Outcomes	Incidence (%)	Inappropriate treatment (%)	Appropriate treatment (%)	OR	95% CI	P-value
CDI resolution [1] (60 cases)	35/60 (58)	11/35 (31)	24/35 (69)	6.91	2.16–22.10	0.001
30-Day all-cause mortality (60 cases)	14/60 (23)	12/14 (86)	2/14 (14)	9.33	1.87–46.69	0.007
30-Day readmission (37 cases)	8/37 (22)	3/8 (37)	5/8 (63)	0.88	0.18–4.24	0.874
8-Week recurrence (39 cases)	7/39 (18)	3/7 (43)	4/7 (57)	0.97	0.19–4.93	0.971

Incidence of outcomes based on whether inappropriate or appropriate treatment was provided. Patients who received appropriate treatment had statistically more (p = 0.001) cases of CDI resolved (OR = 6.91) ,whereas those who received inappropriate treatment had statistically more (p = 0.007) cases of 30-day all-cause mortality (OR = 9.33). Patients who received appropriate treatment also had a greater percentage of 30-day admission (OR = 0.88) and 8-week recurrence (OR = 0.97); however, its occurrence was not statistically different from the inappropriate treatment arm (p = 0.874, 0.971

**Fig. 3:**
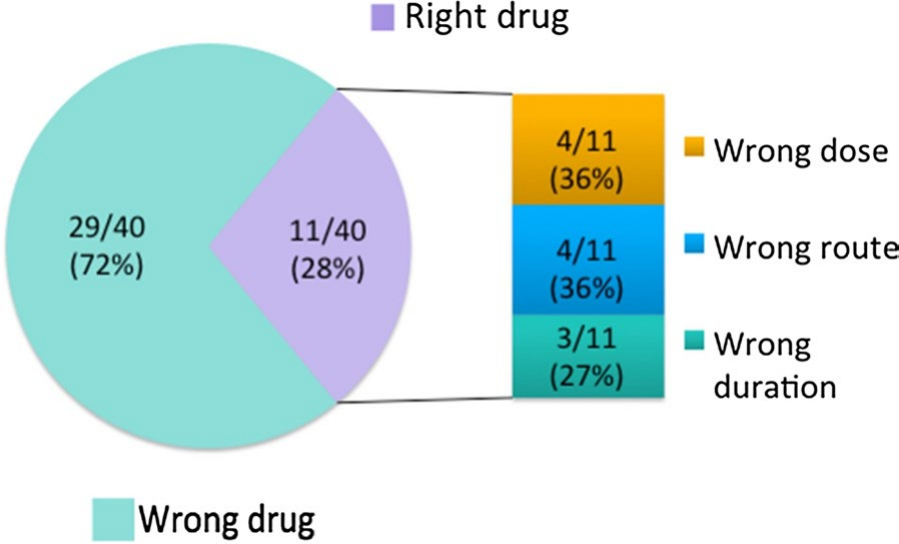
Components of inappropriate treatment. The majority of cases that were treated inappropriately was largely due to the wrong drug prescribed (72%). Of the remaining cases (28%), the correct drug was provided; however, the dose, route, or duration was not appropriate

**Fig. 4:**
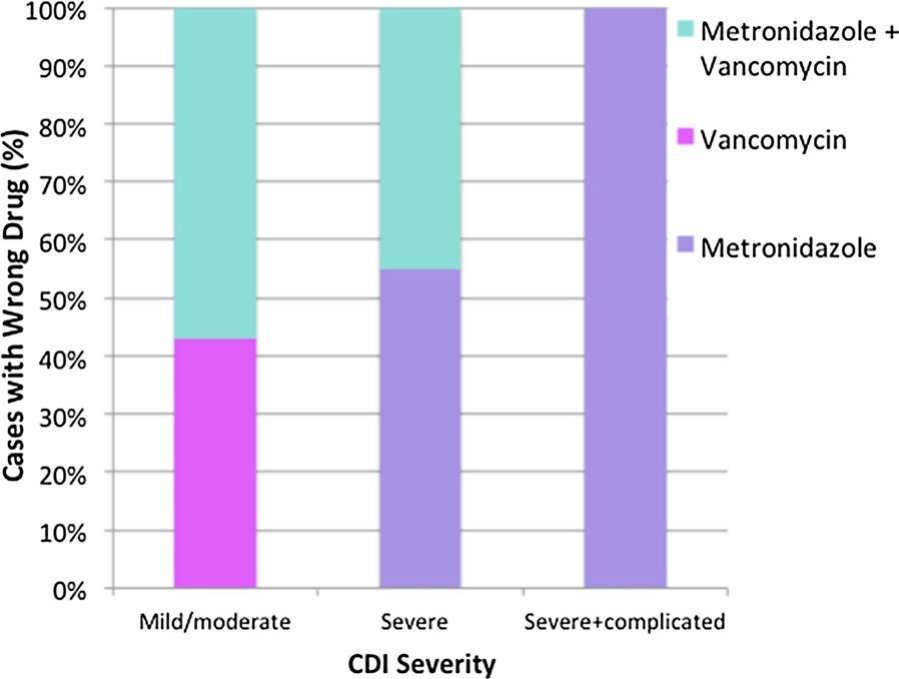
Components of wrong drug by severity. Based on severity, inappropriate drug usage varies. In mild/moderate cases, combination therapy involving metronidazole and vancomycin was incorrectly given the most often. The remaining cases involved vancomycin. In severe cases, inappropriate combination therapy use slightly decreased and metronidazole becomes the most frequently inappropriately given medication. All severe and complicated cases given the wrong drug involved metronidazole

Figure 3 shows the components making up inappropriate CDI treatment, where wrong drug was the highest contributing factor at 72% of cases. Other causes such as wrong dose, route or duration only comprise a total of 28%. Treatment with the wrong drugs can result in suboptimal regimens leading to under treatment or increased risk of recurrence or over treatment and an increased risk of adverse effects and hospital medication costs. Figure 4 further breaks down wrong drugs prescribed per disease severity. Mild-to-moderate cases had a fairly even split of treatment with vancomycin monotherapy or combination therapy. This is inappropriate in this severity as typically only metronidazole monotherapy is required. In severe disease, which had the highest percentage of wrong drug, more than half were prescribed metronidazole monotherapy at 55% and the rest were prescribed combination therapy. Vancomycin is the treatment of choice in these cases and under treatment with metronidazole alone may put the patient at increased adverse outcomes associated with CDI.

**Fig. 5:**
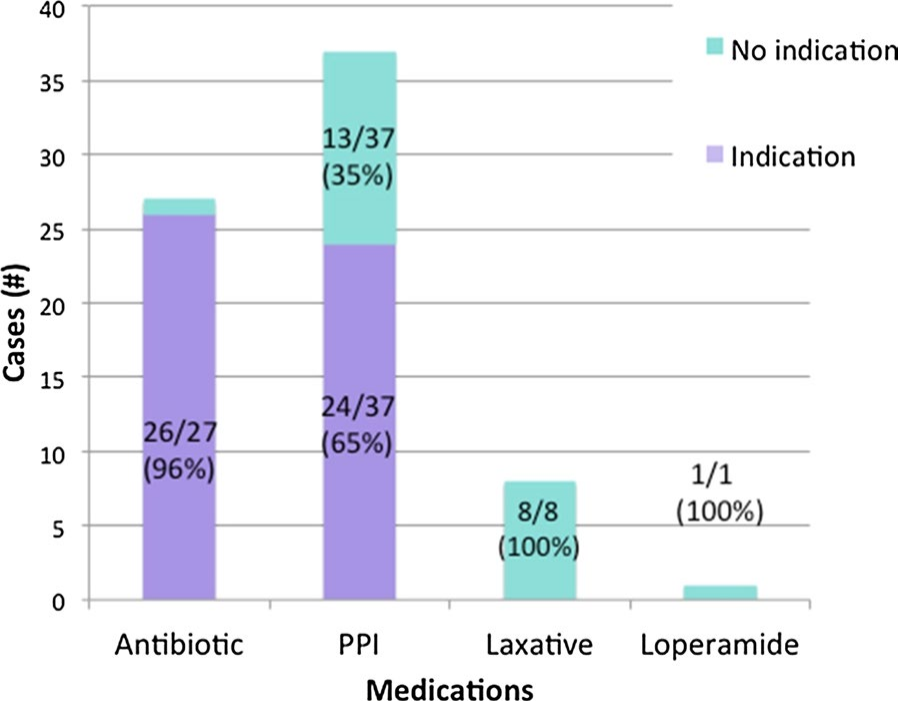
Indications for medications used during CDI. Of the medications used during CDI, most patients receiving antibiotics had an indication, whereas a small percentage of patients did not have an indication. PPIs had a large proportion of cases in which they were not indicated (35%). Patients who received laxatives and loperamide did not have an indication for these medications

The study also looked at the use of other medications known to increase CDI risk such as concurrent antibiotics and PPIs (Fig. 5). About half of patients were prescribed a non-CDI antibiotic and ongoing PPI at the time of diagnosis. However, 96% of the patients on non-CDI antibiotics had a clear indication in which 19% were subsequently discontinued after reassessment. Since many patients are admitted to hospitals for infections, antibiotics are common. This, along with other aforementioned risk factors such as the environment, decreased immune system and exposure to *C. difficile* bacteria create the ideal host for infection. At the time of CDI diagnosis, certain infections still need to be treated with the antibiotic that contributed to the CDI. Therefore, weighing the risks vs. benefits is appropriate to continue non-CDI antibiotic and CDI antibiotics concurrently. Regardless of the situation, non-CDI antibiotics should always be reassessed depending on infection severity as well as the possibility for discontinuation as seen with the 19% antibiotics following CDI diagnosis. PPIs had a much lower rate of indication at 35% in the 62% patients on ongoing PPIs. There are certain indications that require their use indefinitely, and many who are initiated on PPIs, remain on them without proper follow up. A reassessment of PPI use is warranted in those who develop CDI to determine if tapering or discontinuation is possibly. Other medications recommended for discontinuation at the onset of CDI include laxatives and anti-diarrheal agents. The study found that 13% of ongoing laxatives and 2% of loperamide were not discontinued. Whether they were actually given is unclear, since we did not have access to all medication administration records (MARs); however, points at simple areas of improvement for CDI treatment.

In seven cases, CDI antibiotics were continued past the use of non-CDI antibiotics used to treat other infections (Fig. 5). Continuation ranged from 3 up to 15 days with a mean of 7 days. Out of these cases, three recurrent CDIs were identified. There is not enough data to accurately describe the current practice of extending therapy at WDMH or to appropriately describe the rate of recurrence associated with the duration of CDI therapy continuation. Further studies with larger sample size are needed to accurately determine correlation. The time to antibiotic use was also measured in this study, and from the 34 cases that identified times of when patients received antibiotics vs. when they were prescribed varied from immediately up to 11 h. Based on these cases, the time patients received their CDI antibiotics after the prescription was written by a physician ranged from ten minutes up to 11 h, with a mean time of 4:50 ± 2:42 h. CDI as with other infections is a serious infection and patients should receive their antibiotics as soon as possible rather than waiting for the next scheduled time for antibiotic dosing. The longer time to treatment puts patients at risk of adverse outcomes associated with CDI that can be minimized by prompt antibiotic administration.

## Discussion

At WDMH, adherence to the CDI treatment algorithm decreases as the severity of the disease progresses. Similar studies have been published following institutional adherence to IDSA treatment guidelines for CDI at different settings across the US and in Japan. The results of these studies mirror the results seen in this study, where adherence of guidelines for mild-to-moderate cases of CDI, although not particularly high, is still significantly higher than adherence for cases classified as more severe or severe and complicated [14–16]. Although most of these studies are single centre, the study originating in Japan observed the same adherence differences across multiple institutions [17]. Similar patient outcomes were also observed in which a lack of adherence to guidelines resulted in higher rates of mortality compared to patients who received treatment adherent to those recommended in guidelines [18]. Based on these findings at WDMH and these other institutions, this is an important area that can be addressed to improve patient care outcomes.

It is possible that non-adherence may be due to misclassification of severity or a lack of familiarity of the WDMH treatment algorithm. Opportunities for education may be of benefit such as grand round teachings about CDI diagnosis and treatment or an audit and feedback of antibiotics prescribed specifically to treat CDI. A study conducted by Attaar et al. demonstrated that implementation of an electronic order set enhanced clinician adherence to guidelines. Depending on the institution and its available infrastructure, it may not be possible to incorporate this intervention electronically, but it can still be an important avenue to keep in mind. Creating a CDI-based order set may also potentially help streamline the treatment process and improve time to treatment for these patients. This study unveiled an average of almost 5 h in order for confirmed CDI patients to receive treatment. Even if the severity of CDI is rated as mild to moderate, it is still a serious infection in which patients should receive their dose as soon as possible. As the time to treatment increases, so do the adverse outcomes associated with the infection.

Other researchers have also suggested that the disproportionate misuse of metronidazole to treat severe and severe and complicated cases of CDI may stem from both the high cost of vancomycin and the fear of its microbial resistance [15]. Ensuring that antibiotic stewardship interventions in other areas of the hospital or institution are in place can not only help minimize antibiotic-related costs but also minimize overall resistance to ease a fear of using vancomycin. There are also some concerns in prescribing vancomycin to elderly patients with poor renal function; however, oral vancomycin is not systemically absorbed.

Another identified area of improvement is the deprescription of medications often associated with CDI in those with risk factor such as PPIs, laxatives and loperamide. Education surrounding the reassessment of these medications, especially in the presence of other CDI risk factors and in the absence of an indication is important. PPIs are commonly initiated for conditions such as GERD; however, treatment is only intended for the short term in these situations and only patients with a specific indication for ongoing PPIs should be taking these medications long term. There are deprescribing tools that can be made available to clinicians to help educate and guide how to appropriately approach a taper or discontinuation of long-term PPIs.

Data from the study was insufficient to conclude whether or not an extension of CDI treatment after completion of non-CDI antibiotics for other infections reduces CDI relapse. There is still a sparsity of evidence on whether relapse can be prevented or not by extending treatment. Some studies show that there is no difference in relapse rates between these two groups; however, more robust studies are required [19, 20].

The strengths associated with this study are that the results are relevant to our institution, since it can be used for knowledge translation within our institution. In contrast, the limitations associated with this study included it being a retrospective data collection from chart reviews; however, this design was chosen because of time and resource limitations. Therefore, some information was unclear due to the fact that all data were dependent on documentation completed by other health care professionals and were missing. As a result, documentation bias was possible. This was evident in which 26 MARs were missing for 26 CDI cases. Secondly, patients who developed previous or recurrent episode of CDI and were admitted at other institutions were not captured. Thirdly, all information collected and analyzed were limited to the sample size cases limited to the period from June 1, 2017 to June 30, 2018 [21–23].

## Conclusion

In conclusion, an area for improvement in CDI treatment adherence at WDMH is prescribing the right drug based on the disease severity. Specifically in severe CDI, an emphasis is to change therapy to vancomycin alone; in mild-to-moderate CDI, an emphasis is to change therapy to metronidazole alone. Finally in severe and complicated CDI, an emphasis is to change therapy for dual therapy of metronidazole and vancomycin. PPI use in CDI is a common modifiable risk factor commonly being taken in our population during their CDI. As with all PPI use, and especially a concern in our patients, their use should be reassessed for tapering and discontinuation when appropriate. Lastly, treatment with CDI antibiotics should be prescribed and given to patients as soon as possible after confirmation of CDI diagnosis without any delay.

## Data Availability

Data sharing does not apply to this article as no datasets were generated or analyzed during the current study.
